# Influence of Mulberry Leaf Powder Additive on Chemical and Physical Characteristics of Wheat and Rice Flour Butter Cookies

**DOI:** 10.3390/foods13111737

**Published:** 2024-06-01

**Authors:** Dovilė Levickienė, Jurgita Kulaitienė, Nijolė Vaitkevičienė, Laura Rakauskaitė

**Affiliations:** Department of Plant Biology and Food Sciences, Agriculture Academy, Vytautas Magnus University, Donelaicio St. 58, 44248 Kaunas, Lithuania; jurgita.kulaitiene@vdu.lt (J.K.); nijole.vaitkeviciene@vdu.lt (N.V.); laura.rakauskaite@stud.vdu.lt (L.R.)

**Keywords:** butter cookies, mulberry leaves, antioxidant activity, phenolics, phosphorus, texture

## Abstract

People of all age groups consume cookies every day. Consumers’ preferences for cookies supplemented with functional plant raw materials have recently increased. Therefore, this research aimed to investigate the influence of a mulberry leaf additive on the proximate and mineral compositions, total phenolic and total chlorophyll content, antioxidant activity, and the hardness and color properties of butter cookies. Wheat and rice flour butter cookies were prepared by replacing the flour with mulberry leaf powder at 0, 4, 8, and 12% (*w*/*w*). The results revealed that the investigated chemical and physical characteristics of butter cookies depend on the flour used (rice or wheat) and the addition of mulberry leaf powder. Wheat and rice flour cookies with 12% mulberry leaf powder had the significantly highest contents of fiber (20.34 and 20.23%, respectively), ash (1.73 and 1.75%, respectively), K (170.22 and 160.22 mg 100 g^−1^, respectively), and Ca (170.45 and 160.68 mg 100 g^−1^, respectively). The rice flour cookies enriched with 12% leaf powder had the greatest amounts of total phenolics (1.48 mg 100 g^−1^), Zn (12.25 mg kg^−1^), Mn (6.28 mg kg^−1^), Cu (1.95 mg kg^−1^), and antioxidant activity (67.98%). However, the wheat cookies without mulberry leaf powder contained the most B (9.12 mg kg^−1^), while the no-added rice cookies contained the most Fe (14.30 mg kg^−1^). Replacing flour with leaf powder increased the cookies’ hardness and decreased their lightness. In conclusion, enriching butter cookies with freeze-dried mulberry leaves can improve their nutritional value and antioxidant activity.

## 1. Introduction

Cookies are a popular bakery snack and, for the majority of people, are regarded as an important source of energy [1]. They are available in different flavors, have a longer shelf life, and can be obtained in different sorts [2,3,4].

In recent years, there has been an increase in consumer interest in wheat (*Triticum aestivum*)-free diets to reduce the risk of celiac disease, a relatively unknown ailment [5]. However, more people cannot use traditional wheat cookies due to gluten; as a result, the experiment also produced gluten-free rice flour cookies, which could be a good replacement for wheat flour. As a result, gluten-free cereal products have gained popularity in recent decades among gluten-free diet-restricted populations [6]. According to Gujral and Rosell (2004) [7], rice flour is commonly accepted as the best component for gluten-free compositions due to its mild flavor, hypoallergenic qualities, colorlessness, and easily digested carbohydrates. Wheat flour is the base material of cookies and is a good source of carbohydrates; however, it does not contain enough minerals, fiber, or biomolecules like antioxidants [8,9], as well as rice flour [10]. Cookies can be supplemented with various raw materials to increase their nutritional value and health benefits. In addition, various raw material additions may provide a range of tastes, textures, and nutrient benefits in cookies, allowing customers to enjoy a tasty treat while also incorporating healthy foods into their diet.

The mulberry tree (*Morus alba* L.) is a member of the family *Moraceae* and genus *Morus*, which is prevalent in China, Japan, India, and Korea [11], and can thrive in a variety of climatic conditions [12]. It is known as a medicinal plant that protects against various diseases [13]. In Chinese medicine, the leaves and bark of the white mulberry tree are used as a treatment for asthma, bronchitis, insomnia, diabetes, flu, eye infections, and colds [14]. Flavonoids of mulberry leaves such as quercetin, rutin, and isoquercitrin, scavenge free radicals, indicating possible anti-oxidant activity. The presence of prenylated flavonoids enhanced its antioxidant claims. Furthermore, these antioxidants offer cardiovascular protection by inhibiting LDL oxidation and hence atherosclerosis [15]. Studies [15,16,17] have reported that mulberry leaves are rich in carbohydrates, proteins, fats, and vitamins. Mulberry has been found to possess a high concentration of phenolic compounds such as rutin, astragalin, and chlorogenic acid [18,19], which are proven to have biological functions such as anticancer, anti-aging, anti-obesity, and anti-diabetes effects [20,21,22,23]. In addition, white mulberry leaves contain a wide range of micro and macro elements [24,25].

Mulberry has been recognized as a functional food due to its high nutritional and phytochemical content [15] and is beneficial for human health [26]. The leaves are used as dairy animal feed, teas [27,28], as an additive in yogurt bites [29], jelly [30], masala biscuits [31], wheat flour cookies [32], noodles [33], and salad ingredients. Because of mulberry leaves’ medical and nutritional benefits, leaf powder may be used as a source of nutrients in food products to supplement nutritional value.

As well as sourcing mulberry leaves from local suppliers, this reduces the environmental impact of transportation and supports the local economy. There has been little research on the chemical composition (proximate composition, macro- and microelements, and total phenolic and chlorophyll contents) of cookies enriched with mulberry leaves, and most of the studies carried out have focused on physical characteristics only (color parameters, hardness, DPPH free-radical scavenging activity, and sensory properties) [6]. In addition, there are no studies on cookies baked with rice flour and enriched with mulberry leaves. Therefore, this study aims to investigate the influence of cultivar ‘Galicia’ mulberry leaf powder additive on the chemical and physical parameters of wheat and rice flour butter cookies.

## 2. Materials and Methods

### 2.1. Materials

The ingredients of the butter cookies: white wheat flour (550 C type, origin country Lithuania), rice flour (origin country Pakistan), butter (82% fat) (origin country Lithuania), eggs (origin country Lithuania), sugar (origin country Lithuania), and baking powder (origin country Poland) were purchased from the local market in Kaunas. The Vytautas Magnus University Academy of Agriculture, Faculty of Agronomy, Department of Plant Biology and Food Sciences, Laboratory of Food Raw Materials Processing, prepared the cookies. Table 1 shows the nutritional value of white wheat flour and rice flour, according to the manufacturer’s data.

The white mulberry (*Morus alba* L.) leaf powder was used to enrich the butter cookies. White mulberry of cultivar ‘Galicia’ was collected in 2022 on a farm in the Kaunas district, Lithuania (54°53′50″ N 23°53′10″ E). The leaves were washed, drained, frozen at −35 °C, then lyophilized using a Freeze-Drying Plant Sublimator 3 × 4 × 5 (ZIRBUS Technology GmbH, Bad Grund, Germany) and ground to a fine powder for 1 min at 8000 rpm using a laboratory mill (Grindomix GM 200, Retsch GmbH, Haan, Germany). Table 2 shows the chemical composition of mulberry leaves.

### 2.2. Preparation of the Butter Cookies

A two-factor experiment was performed. Factor A: type of flour: 1. wheat flour; and 2. rice flour. Factor B: different amounts of mulberry leaf additive: (1) 0% (control); (2) 4%; (3) 8%; and (4) 12%. To improve the nutritional value of the butter cookies, mulberry leaf powder amounts (0, 4, 8, and 12%) were used to replace the percentage of wheat and rice flour. The experiment was performed with four replicates for each treatment. Table 3 (for one replicate) shows the recipe for cookies enriched with different amounts of mulberry leaf powder.

The ingredients, such as butter, eggs, and sugar, were mixed using a mixer until they formed a cream. Then, we added wheat or rice flour and an appropriate amount of mulberry leaf powder and mixed for 4 min. The dough was refrigerated at 6 °C for 30 min. To make cookies, the dough was sheeted with a roller to a thickness of 4 mm, cut with a circular cookie cutter with a diameter of 5 cm, placed on a baking tray with baking paper, and then baked at 170 °C for 10 min in a preheated oven. Baked cookies were cooled down to room temperature for 2 h, packed in sealed plastic bags, and stored until analysis.

### 2.3. Proximate Analysis

The moisture in freeze-dried white mulberry leaves and butter cookie samples was determined by drying samples at 105 °C to a constant weight [34], and the amount of fiber by using the Association of Official Agricultural Chemists (AOAC) official methods, the amount of ash by using combustion at 550 °C [35], the amount of protein by the Kjeldahl method using KJELDATHERM (Gerhardt, Königswinter, Germany), and the pH by using a pH meter (MeterLab PHM210, Villeurbanne, France) [36].

### 2.4. Determination of Chlorophyll Content

The contents of chlorophyll a and chlorophyll b in mulberry leaves and cookies were determined spectrophotometrically using a spectrometer Labomed Inc., Los Angeles, CA, USA, at the wavelengths 662 nm and 644 nm [37]. In total, 1 g of samples were homogenized and extracted with acetone for 15 min using a magnetic stirrer at 700 rpm. Results were expressed in mg 100 g^−1^.

### 2.5. Macro- and Microelement Analysis

The elements of phosphorus (P), potassium (K), calcium (Ca), iron (Fe), magnesium (Mg), copper (Cu), boron (B), zinc (Zn), and manganese (Mn) were measured in butter cookies. These elements were identified using inductively coupled plasma atomic emission spectrometry (ICP-AES) [38]. Before ICP-AES testing, butter cookies samples were treated with hydrogen peroxide and nitric acid at 100 °C until digestion was finished. A total of 1 g of butter cookie sample was mixed with known quantities of hydrogen peroxide and nitric acid and heated. The resulting solution was analyzed using an Optima 2100 DV atomic-emission spectrometer (Perkin Elmer, Waltham, MA, USA) after digestion. Within the concentration range of the mineral components in the cookie samples, the proper standards for each mineral were established. In the same circumstances as the samples, standard blank solutions were produced. All chemical analyses were performed in triplicate. P, K, Ca, and Mg results were expressed in mg 100 g^−1^ DM, as well as Zn, Mn, Fe, B, and Cu in mg kg^−1^ DM.

### 2.6. Total Phenolic Amount Analysis

The total phenolic amount of butter cookies was determined by using the Folin–Cocalteu spectrophotometric method, as reported by Tamilselvi et al. [39]. A total of 1.0 g of butter cookies was mixed with 10 mL of 70% ethanol and extracted for 30 min in an ultrasonic laboratory bath AU-65 (Argolab, Torino, Italy). Then, the extract was centrifuged for 30 min at 3000 rpm using centrifuge Rotofix 32A (ANDREAS hettich GmbH and Co., Tuttlingen, Germany). The obtained extract was then combined with 1 mL of sodium carbonate (20%), 0.2 mL of the Folin–Ciocalteu reagent, and 5 mL of pure water. To measure the absorbance at 760 nm after 30 min of incubation at 20 °C in the dark, a spectrophotometer (Labomed Inc., Los Angeles, CA, USA) was used. The total phenolic amount was calculated with the calibration curve using gallic acid equivalent standards. The results for the total phenolic content were expressed as mg of gallic acid equivalent (GAE) per g of DM.

### 2.7. Determination of Antioxidant Activity

According to the method described by Leong and Shui [40] with some modifications, the DPPH free-radical scavenging activity of each sample was assessed using the Ultraspec 3000 UV/vis Spectrophotometer (Labomed Inc., Los Angeles, CA, USA). DPPH solution in methanol at 0.1 mM was prepared. The initial absorbance of the DPPH in methanol was measured at 515 nm and did not change throughout the assay. After 30 min, the change in absorbance at 515 nm was measured. An aliquot of 0.4 mL of an extract was added to 3 mL of methanolic DPPH solution. Results were expressed as the percentage (%) of DPPH binding capacity.

### 2.8. Color Parameter Analysis

The color parameters L* (lightness), a* (positive-red, negative-green), and b* (positive-yellow, negative-blue, NBS units) of a butter cookie were measured using a spectrophotometer ColorFlex (Hunter Associates Laboratory, Inc., Plainfield, IL, USA). All analyses were performed in four replicated. Ten cookies of each treatment were milled and analyzed.

### 2.9. The Hardness of Butter Cookies

A texture profile analysis (TPA) of the butter cookies samples was carried out using the analyzer TA. XT Plus (Stable Micro Systems, Surrey, UK) was equipped with a P20 adapter moving at a rate of 3 mm/s, and the penetration depth into cookie samples was 5 mm. The maximum force (the hardness (N)) opposed to fracture was measured [41]. Ten cookies of each treatment were analyzed.

### 2.10. Statistical Analysis

A two-way analysis of variance (ANOVA) method was used to statistically process all data with the software program STATISTIKA (Statistica 10; StatSoft, Inc., Tulsa, OK, USA). A Tukey HSD test (*p* < 0.05) was performed to determine the statistical significance of the differences between the means.

## 3. Result and Discussion

### 3.1. Proximate Composition of Butter Cookies

The moisture content ranged from 3.19 to 4.54%; wheat flour cookies with 4% mulberry leaf additive had the highest moisture content, while wheat flour cookies with 8% mulberry leaf additive had the lowest (3.19%) (Table 4). Andresen et al. [42] investigated the effect of baking time and temperature on moisture content in butter cookies and found that the contents of this parameter ranged from 1.1% to 9.4%. According to the literature, a high moisture content reduces the shelf life of cookies and causes a delay in the caramelization and Maillard reactions [43].

A higher content of protein was found in all treatments of wheat flour cookies (from 5.75% to 6.77%), compared with rice flour cookies (from 2.96% to 4.76%) (Table 4). The replacement of wheat and rice flour with mulberry leaf powder had a significant effect on the protein content. Wheat flour cookies with 8% and 12% mulberry leaf additive had the significantly highest amount of this compound, while rice cookies without mulberry leaf additive had the significantly lowest amount. Wheat flour is a high-protein food that contains more protein than white rice [44]. Tian et al. [44] reported that the protein content of different rice flours was 27.24–43.04% lower than that of common wheat. Mulberry leaf powder is also high in protein, with levels ranging from 18.41 to 24.63% depending on genotypes [45].

With increasing amounts of mulberry leaf additive, the quantities of fiber and ash in both wheat and rice cookies significantly increased. Replacing wheat flour with 4–12% mulberry leaf additives led to an increase in fiber from 13.80% to 62.20% and in ash from 51.06% to 84.04%. Compared with rice cookies without additive (control), the content of ash in rice cookies with 4–12% mulberry leaf additive increased from 22.56% to 87.84%, and the content of fiber increased from 16.81% to 47.06%. Wheat and rice cookies with a 12% mulberry leaf additive had significantly higher contents of these compounds compared to other cookie samples. According to the literature, in dried mulberry leaves, ash ranged from 14.59 to 17.24% [15] and fiber ranged from 11.46 to 16.61% [28]. Therefore, we can use mulberry leaf additives to increase the amounts of the mentioned compounds in various types of food products. The pH values of the butter cookies varied from 6.58 to 7.57. The pH values of the wheat and rice cookies increased as the added amounts of mulberry leaf increased. The maximum values of this parameter were found in the wheat and rice cookies with a 12% mulberry leaf additive (7.57 and 7.50, respectively).

Sayuti et al. [32] evaluated the nutritional value of wheat flour cookies containing mulberry leaf (10, 20, and 30%) and mulberry leaf extract (10, 20, and 30%). It was discovered that increasing the amounts of mulberry leaf and extract in cookies increased the ash and protein content. This could be due to the high mineral and protein content of mulberry leaves, according to the authors. Makchuay et al. [33] investigated the effect of mulberry leaf tea powder on the protein and fiber content of rice noodles. The addition of powder at 5 to 25% increased crude fiber content by 5–8 times and the protein content of noodles by 1.16–1.55 times compared with rice noodles without additives.

### 3.2. Mineral Elements Evaluation of Butter Cookies

The contents of macroelements such as P, K, Ca, and Mg varied significantly depending on the flour used and the addition of mulberry leaf powder. P was the most abundant element in all of the cookie samples tested, followed by K, Ca, and Mg (Table 5). All rice flour cookie samples demonstrated 8.37–30.71% higher P values when compared to wheat flour cookies. We found the highest concentrations of this mineral in rice flour cookies containing 4%, 8%, and 12% mulberry leaf additive (380.47, 390.82, and 390.91 mg 100 g^−1^, respectively), with no significant difference between them.

The addition of mulberry leaf powder as an additive also increased the contents of K and Ca in butter cookies. Both the wheat and rice cookies with a 12% mulberry leaf additive had the significantly highest amounts of K (170.22 and 160.22 mg 100 g^−1^, respectively) and Ca (170.45 and 160.68 mg 100 g^−1^, respectively), while the control wheat and rice cookie samples recorded the lowest contents of these minerals. All treatments of rice flour cookies showed a higher Mg content (from 24.91 to 42.33 mg 100 g^−1^) compared to wheat flour cookies (from 13.24 to 32.21 mg 100 g^−1^). Rice flour cookies with 8% and 12% mulberry leaf additive had the highest content of 36.21 and 42.33 mg 100 g^−1^, respectively, while wheat flour cookies without additive had the lowest content of 13.24%.

According to the data of this study, the freeze-dried mulberry leaf additive increases the contents of the analyzed macroelements in cookies due to the high amount of these elements in the leaves, which is much higher than in wheat or rice flour. Mulberry leaf powder is an excellent source of some important minerals, particularly calcium (2870.08 mg 100 g^−1^), potassium (1630.14 mg 100 g^−1^), phosphorus (430.88 mg 100 g^−1^), and magnesium (270.68 mg 100 g^−1^) [29]. However, in the scientific literature, there is no information about the mineral element contents of butter cookies enriched with mulberry leaf powder. Kim et al. [30] evaluated the macroelement content of jelly with various levels of mulberry leaf powder (0.5, 1, 1.5, and 2%). They revealed that increasing the amount of mulberry leaf powder added significantly increased the contents of K, Ca, and Mg. In a study conducted by Kulaitienė et al. [29], yogurt bites with 1% mulberry leaf powder contained 41.24% significantly higher amounts of K, 21.14% more Ca, 38,08% more P, and 139.70% more Mg than the yogurt bites without additives.

The amounts of Fe, Zn, Mn, Cu, and B in the cookie samples also varied depending on the flour used and the amount of mulberry leaf additive (Table 6). The Fe content in rice flour cookies (control) was significantly (64.37%) higher than in wheat flour cookies (control). In wheat cookies with a 12% mulberry leaf additive, the mineral content increased by 12.41% compared to control wheat cookies. However, the replacement of rice flour with mulberry leaf powder had a negative effect on the Fe content of rice flour cookies. Zn content was lower in wheat flour cookies (from 5.28 to 6.02 mg kg^−1^) than in rice flour cookies (from 9.88 to 12.25 mg kg^−1^). In wheat flour cookies, the mulberry leaf additive did not significantly influence the Zn content. However, rice flour cookies with 12% powder contained the significantly highest amount of this element among the rice cookie samples. The mulberry leaf supplementation had a negative effect on the cookie’s B content. The significantly highest content of B was in the control wheat flour cookies (9.12 mg kg^−1^). The Mn and Cu content in rice flour cookies were also higher than those in wheat flour cookies. Mn varied from 1.95 to 3.40 mg kg^−1^ and Cu from 0.73 to 1.00 mg kg^−1^ in wheat flour cookies. In rice flour cookies, Mn ranged from 5.25 to 6.28 mg kg^−1^, and Cu from 1.65 to 1.95 mg kg^−1^. The content of these minerals in both wheat and rice cookies increased with increasing levels of mulberry leaf powder. Wheat and rice flour cookies with a 12% leaf additive showed significantly higher content of Mn and Cu, while wheat and rice flour cookies without the additive had the lowest.

Our results are consistent with previous researchers’ findings that the incorporation of mulberry leaf powder in food products increases some microelement content. The addition of mulberry leaf powders to the formulation of yogurt bites allowed for a significant increase in the amount of Fe, Zn, and Mn in the final products [29]. Wheat flour masala biscuits enriched with 7.5% mulberry leaf powder contained 3.75 mg zinc, 19.66 mg iron, 3.19 mg manganese, and 5.68 mg copper, while control biscuits without mulberry additive had 2.34 mg zinc, 19.31 mg iron, 2.06 mg manganese, and 3.65 mg copper [31]. Also, our results showed that rice is a great gluten-free alternative to wheat flour. Rice has higher levels of Fe, Zn, Mn, and Cu than wheat, making it suitable for processing into flour and use in various beneficial food products [46,47].

### 3.3. Evaluation of Total Chlorophyll, Phenolic Amount, and Antioxidant Activity in Butter Cookies

**Figure 1:**
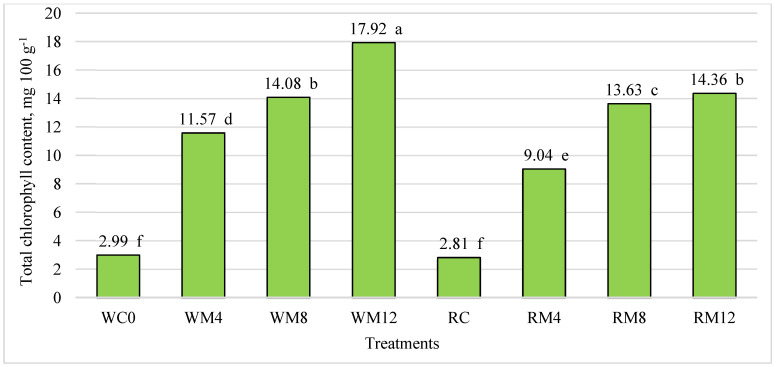
Influence of mulberry leaf additive on the total chlorophyll content of butter cookies, mg 100 g^−1^. The differences between the means not marked by the same letter (a, b, c, d, e, f) are significant at *p* ≤ 0.05. WC—wheat flour cookies without mulberry leaf additive (control), WM4—wheat flour cookies with 4% mulberry leaf additive, WM8—wheat flour cookies with 8% mulberry leaf additive, WM12—wheat flour cookies with 12% mulberry leaf additive, RC—rice cookies without mulberry leaf additive (control), RM4—rice flour cookies with 4% mulberry leaf additive, RM8—rice flour cookies with 8% mulberry leaf additive, RM12—rice flour cookies with 12% mulberry leaf additive.

The conducted studies showed that the total amount of total chlorophyll in butter cookies ranged from 2.81 to 17.92 mg 100 g^−1^ (Figure 1). The results showed that adding mulberry leaf powder to butter cookies increased the amount of total chlorophyll. Wheat flour cookies with a 12% mulberry leaf additive showed six times a significantly higher amount of total chlorophyll compared to wheat cookies without the additive. Cookies made from wheat and rice flour without the additive had the significantly lowest amount of total chlorophyll.

Other researchers conducted a study on semolina pasta enriched with 0, 1, 2, 3, 4, and 5% nettle leaf supplement. The study showed that the lowest total chlorophyll content of 0.98 mg 100 g^−1^ was found in the no-added noodles, while the highest total chlorophyll content was found to be 23.76 mg 100 g^−1^ with the additive [48].

**Figure 2:**
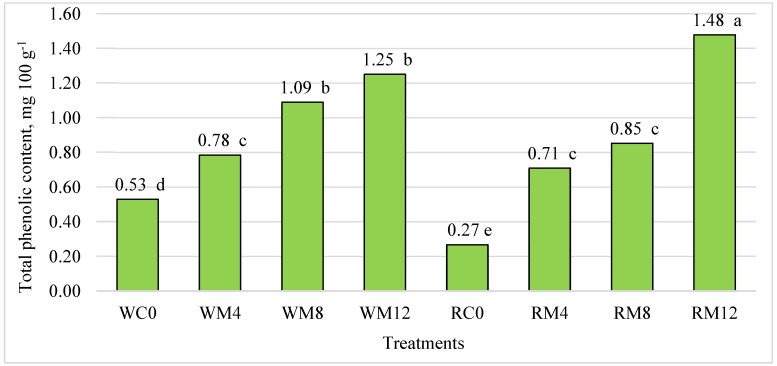
Influence of mulberry leaf additive on the total phenolic content of butter cookies, mg 100 g^−1^. The differences between the means not marked by the same letter (a, b, c, d, e) are significant at *p* ≤ 0.05. WC—wheat flour cookies without mulberry leaf additive (control), WM4—wheat flour cookies with 4% mulberry leaf additive, WM8—wheat flour cookies with 8% mulberry leaf additive, WM12—wheat flour cookies with 12% mulberry leaf additive, RC—rice cookies without mulberry leaf additive (control), RM4—rice flour cookies with 4% mulberry leaf additive, RM8—rice flour cookies with 8% mulberry leaf additive, RM12—rice flour cookies with 12% mulberry leaf additive.

Our studies showed that the phenolic content of the butter cookies ranged from 0.27 to 1.48 mg 100 g^−1^ (Figure 2). In both types of cookies, the addition of mulberry leaves significantly increased the content of phenolic compounds compared to the control. We found that the addition of 4, 8, and 12% mulberry leaf to both wheat and rice flour cookies significantly increased the content of phenolic compounds by 1.47, 2.1, 2.36, 2.62, 3.15, and 5.48 times, respectively, compared to the control treatment.

A comparison between wheat and rice flour cookies without additives showed significant differences. The wheat flour cookies were found to have a higher content of phenolic compounds, by as much as 96.30%. According to the literature, the addition of mulberry leaf powder to yogurt bites [29], snacks [49], and rice noodles [33] significantly increased the total phenolic compound content. 

Other scientists investigated that the addition of mulberry leaf extract also increased the content of phenolic compounds in bread [50]. 

**Figure 3:**
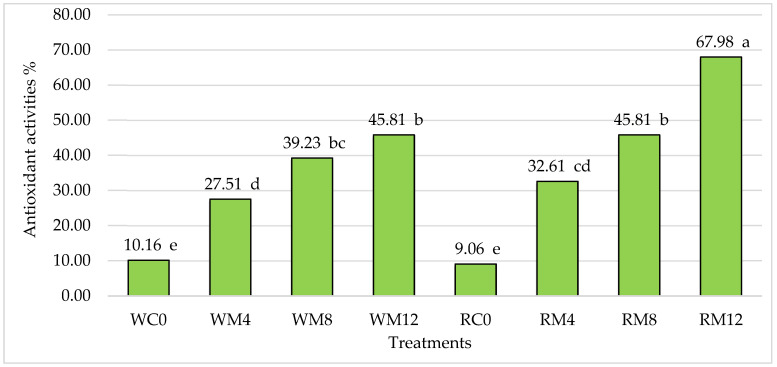
Influence of mulberry leaf additives on antioxidant activities in butter cookies, %. Note: The differences between the means not marked by the same letter (a, b, c, d, e) are significant at *p* ≤ 0.05. WC—wheat flour cookies without mulberry leaf additive (control), WM4—wheat flour cookies with 4% mulberry leaf additive, WM8—wheat flour cookies with 8% mulberry leaf additive, WM12—wheat flour cookies with 12% mulberry leaf additive, RC—rice cookies without mulberry leaf additive (control), RM4—rice flour cookies with 4% mulberry leaf additive, RM8—rice flour cookies with 8% mulberry leaf additive, RM12—rice flour cookies with 12% mulberry leaf additive.

According to M. Polumackanyxz et al. [51], antioxidant activity is an important parameter for determining the health benefits of foods. Our results revealed that the addition of mulberry leaves had a significant effect on the antioxidant activity of the cookies (Figure 3). The antioxidant activity of the butter cookies ranged from 9.06 to 67.98%. The significantly lowest antioxidant activity was in both types of cookies without additives, while the significantly highest was in the rice flour cookies with a 12% additive. The addition of mulberry leaves increased the antioxidant activity of the wheat and rice flour cookies by an average of 3.70 and 5.39 times, respectively, compared to the control cookie samples.

A study by other researchers showed that the cookies with a 7% addition of mulberry leaf contained the highest antioxidant activity (31.11%), while the cookies with no addition had the significantly lowest (7.21%) [6]. Thailand researchers’ results showed that antioxidant activity increased in four snack products (loaf bread, Thai steamed cupcakes, green coconut sweet pudding, and snow skin mooncake) supplemented with 10% mulberry leaf powder. Therefore, the bioactive properties of mulberry leaf powder suggest their possible use for supplementing healthy foods [49].

### 3.4. Physical Characteristics of the Cookies

The texture, as well as the color properties of bakery products, play an important role in consumer acceptability [52,53].

The physical properties of butter cookies, such as hardness and color, were influenced by the addition of mulberry leaves and are summarized in Table 7. A comparison of wheat and rice flour cookies’ L* color coordinates revealed significant differences between all treatments. The addition of mulberry leaf led to a significant darkening of the cookies, with the rice flour cookies containing a 12% addition being the darkest. The control cookies were significantly lighter compared to the cookies with additives.

Assessing the a* value, which indicates the red or green color, it was found that the wheat and rice flour cookies without additives were significantly reddest at 8.76 and 9.36, respectively, while the wheat flour cookies with a 12% addition were characterized by a* negative value (−1.26) (Table 7). The intensity of the red color in wheat and rice flour cookies with additives decreased by an average of 6.95 and 37.44 times, respectively, compared to the control.

Our research revealed that b* color coordinates ranged from 32.49 to 39.80 in wheat flour cookies and 30.59 to 36.47 in rice flour cookies (Table 7). After evaluating the b* value, it was found that the addition of mulberry leaves had a significant effect on the yellow-ness of the cookies, and wheat flour cookies with 8 and 12% mulberry leaves were significantly yellower compared to all treatments of samples.

A researcher Park [6], performed a study where cookies were enriched with 0, 1, 3, and 7% mulberry leaf additive. The study’s result was similar to ours. The control cookies without additives were significantly lighter (75.51 NBS), while cookies with a 7% mulberry leaf additive were significantly darker (51.23 NBS). The significantly highest value of a* coordinate was (2.15) in cookies without additive and the significantly lowest (−1.83) in cookies with 7% mulberry leaf additive. Cookies with 7% mulberry leaf addition showed the significantly highest (40.11) b* value difference, while the control variant showed the lowest (16.10).

The cookies’ hardness varied from 19.96 to 42.70 N, depending on both factors (Table 7). Wheat flour cookies were found to be significantly harder by 1.5 times compared to rice flour cookies. Both wheat and rice flour butter cookies with 12% leaf addition were significantly harder by 42.00 and 96.04% N, respectively, compared to the control. The addition of 4 and 8% mulberry leaves had no significant effect on wheat flour cookies, while this additive significantly increased the hardness by 41.93 and 56.01%, respectively, in rice flour cookies.

In a study conducted by Park [6], wheat flour was replaced with 0, 1, 3, 5, and 7% mulberry leaf additive in cookies, and it was found that the addition of mulberry leaf powder increased hardness.

## 4. Conclusions

Butter cookies enriched with freeze-dried mulberry leaf powder showed higher proximate composition, especially protein, fiber, ash, and chlorophyll content, than the control cookies. The use of 4, 8, and 12% additives in wheat and rice flour cookies significantly increased the amounts of phenolic compounds by 1.47, 2.06, and 2.36, and 2.63, 3.15, and 5.48 times, respectively, as well as antioxidant activity by 2.71, 3.86, and 4.51, and 3.60, 5.06, and 7.50 times, respectively, compared to the control cookies. The physical properties also were affected by the mulberry leaf additive positively by demonstrating cookies’ yellowness, redness, and darkness, as well as increased hardness, leading to the cookies’ better well-acceptability and texture characteristics. According to the findings of the current study, the addition of freeze-dried mulberry leaf powder improved the nutritional value of the butter cookies; therefore, mulberry leaf could be offered to the food industry as a potential source of fiber, phenolic compounds, and minerals.

## Figures and Tables

**Table 1:** Nutrition value of flour, 100 g.

Nutrition Value	White Wheat Flour Type 500 C	Rice Flour
Energy value (kJ/Kcal)	1482.0/350.0	1463.0/345.0
Fat	1.2	1
Of which saturates	0.4	0.2
Carbohydrate	70	76
Of which sugars	0.5	0.5
Fiber	2.9	1
Protein	12	7.4
Salt	0.1	0.01

**Table 2:** The chemical composition of freeze-dried white mulberry leaves.

Composition	Content
Dry matter, %	96.78
Protein, %	14.04
Fiber, %	12.55
Ash, %	11.59
Total chlorophyll content, mg 100 g^−1^	341.05
Total phenolic content, mg 100 g^−1^	28.33
K (mg 100 g^−1^)	1630.14
Ca (mg 100 g^−1^)	2870.08
P (mg 100 g^−1^)	430.88
Mg (mg 100 g^−1^)	270.68
Fe (mg kg^−1^)	7.93
Zn (mg kg^−1^)	2.73
B (mg kg^−1^)	2.71
Mn (mg kg^−1^)	1.82
Cu (mg kg^−1^)	0.75

**Table 3:** Recipe for butter cookies with different amounts of mulberry leaf powder.

Ingredients, g	Mulberry Leaf Powder Content of 0% (Control)	Mulberry Leaf Powder Content of 4%	Mulberry Leaf Powder Content of 8%	Mulberry Leaf Powder Content of 12%
Wheat/rice flour	185	177.6	170.2	162.8
Mulberry leaf powder	0	7.4	14.8	22.2
Sugar	80	80	80	80
Butter	115	115	115	115
Baking powder	5	5	5	5
Egg	50	50	50	50

**Table 4:** Influence of mulberry leaf powder additive on the proximate composition of butter cookies, %.

Treatments	Moisture	Protein	Fiber	Ash	pH
WC	3.88 c	5.75 b	12.54 d	0.94 e	6.81 bc
WM4	4.54 a	6.32 ab	14.27 c	1.42 c	7.02 b
WM8	3.19 e	6.57 a	17.21 b	1.54 b	7.05 b
WM12	3.79 c	6.77 a	20.34 a	1.73 a	7.57 a
RC	3.39 d	2.96 e	10.77 e	1.19 d	6.58 c
RM4	3.85 c	3.68 d	13.20 cd	1.39 c	6.83 bc
RM8	3.47 d	4.40 c	17.13 b	1.57 b	7.03 b
RM12	4.07 b	4.76 c	20.23 a	1.75 a	7.50 a

Note: Different letters in the same column represent significant differences (*p* < 0.05). WC—wheat flour cookies without mulberry leaf additive (control), WM4—wheat flour cookies with 4% mulberry leaf additive, WM8—wheat flour cookies with 8% mulberry leaf additive, WM12—wheat flour cookies with 12% mulberry leaf additive, RC—rice cookies without mulberry leaf additive (control), RM4—rice flour cookies with 4% mulberry leaf additive, RM8—rice flour cookies with 8% mulberry leaf additive, RM12—rice flour cookies with 12% mulberry leaf additive.

**Table 5:** Influence of mulberry leaf powder additive on the contents of macroelements of butter cookies, %.

Treatments	Macroelements (mg 100 g^−1^ DM)			
	Phosphorus (P)	Potassium (K)	Calcium (Ca)	Magnesium (Mg)
WC	260.51 d	110.14 c	29.84 e	13.24 d
WM4	350.45 bc	140.51 b	91.51 c	21.14 c
WM8	350.64 bc	140.91 b	120.15 b	24.64 c
WM12	360.74 b	170.22 a	170.45 a	32.21 b
RC	340.51 c	100.32 c	21.58 e	24.91 c
RM4	380.47 a	140.14 b	72.13 d	31.52 b
RM8	390.82 a	140.61 b	110.56 b	36.21 a
RM12	390.91 a	160.22 a	160.68 a	42.33 a

Note: Different letters in the same column represent significant differences (*p* < 0.05). WC—wheat flour cookies without mulberry leaf additive (control), WM4—wheat flour cookies with 4% mulberry leaf additive, WM8—wheat flour cookies with 8% mulberry leaf additive, WM12—wheat flour cookies with 12% mulberry leaf additive, RC—rice cookies without mulberry leaf additive (control), RM4—rice flour cookies with 4% mulberry leaf additive, RM8—rice flour cookies with 8% mulberry leaf additive, RM12—rice flour cookies with 12% mulberry leaf additive.

**Table 6:** Influence of mulberry leaf powder additive on microelements in butter cookies, %.

Treatments	Microelements (mg kg^−1^ DM)				
	Iron (Fe)	Zinc (Zn)	Boron (B)	Manganese (Mn)	Cooper (Cu)
WC	8.70 c	5.28 c	9.12 a	1.95 e	0.73 e
WM4	8.55 c	5.58 c	5.15 c	2.35 de	0.90 d
WM8	8.70 c	6.02 c	4.78 c	2.83 cd	0.95 d
WM12	9.78 b	5.68 c	5.13 c	3.40 c	1.00 d
RC	14.30 a	9.88 b	7.06 b	5.25 b	1.65 b
RM4	8.93 c	10.20 b	5.30 c	5.78 b	1.40 c
RM8	10.81 b	10.36 b	4.52 c	5.98 b	1.37 c
RM12	9.25 b	12.25 a	5.60 c	6.28 a	1.95 a

Note: Different letters in the same column represent significant differences (*p* < 0.05). WC—wheat flour cookies without mulberry leaf additive (control), WM4—wheat flour cookies with 4% mulberry leaf additive, WM8—wheat flour cookies with 8% mulberry leaf additive, WM12—wheat flour cookies with 12% mulberry leaf additive, RC—rice cookies without mulberry leaf additive (control), RM4—rice flour cookies with 4% mulberry leaf additive, RM8—rice flour cookies with 8% mulberry leaf additive, RM12—rice flour cookies with 12% mulberry leaf additive.

**Table 7:** The influence of mulberry leaf additives on the harness and color characteristics of butter cookies.

Treatments	Hardness (N)	Color		
		L*	a*	b*
WC0	30.07 b	70.96 a	8.76 a	32.49 d
WM4	30.98 b	60.90 c	3.13 b	38.69 b
WM8	33.35 b	55.67 e	0.12 d	39.41 a
WM12	42.70 a	53.44 f	−1.26 e	39.90 a
RC0	19.96 c	68.09 b	9.36 a	30.59 e
RM4	28.33 b	58.94 d	3.85 b	36.14 c
RM8	31.14 b	52.73 f	1.37 c	36.38 c
RM12	39.13 a	49.80 g	0.25 d	36.47 c

Note: Different letters in the same column represent significant differences (*p* < 0.05). WC—wheat flour cookies without mulberry leaf additive (control), WM4—wheat flour cookies with 4% mulberry leaf additive, WM8—wheat flour cookies with 8% mulberry leaf additive, WM12—wheat flour cookies with 12% mulberry leaf additive, RC—rice cookies without mulberry leaf additive (control), RM4—rice flour cookies with 4% mulberry leaf additive, RM8—rice flour cookies with 8% mulberry leaf additive, RM12—rice flour cookies with 12% mulberry leaf additive.

## Data Availability

The original contributions presented in the study are included in the article, further inquiries can be directed to the corresponding author.
